# Good governance, public health expenditures, urbanization and child undernutrition Nexus in Ethiopia: an ecological analysis

**DOI:** 10.1186/s12913-018-3822-2

**Published:** 2019-01-15

**Authors:** Sibhatu Biadgilign, Habtamu Yesigat Ayenew, Arega Shumetie, Stanley Chitekwe, Assaye Tolla, Demewoz Haile, Seifu Hagos Gebreyesus, Amare Deribew, Betemariam Gebre

**Affiliations:** 1 Public Health Nutrition Research Consultant, P.O. Box 24414, Addis Ababa, Ethiopia; 2 Technical University Munich, Munich, Germany; 3 Department of Economics, Haramaya University of Ethiopia, Dire Dawa, Ethiopia; 4 United Nations Children’s Fund (UNICEF), Nepal Country Office UN House, New York, USA; 5 United Nations Children’s Fund (UNICEF), Nigeria Country Office, UN House, Plot 617/618 Central Area District Diplomatic Zone, Garki, P M, Abuja, B 2851 Nigeria; 6 School of Public Health, College of Health Sciences, Addis Ababa University, Addis Ababa, Ethiopia; 7 St. Paul Millennium Medical College, Addis Ababa, Ethiopia; 8 Nutrition International (former Micronutrient Initiative), Ottawa, Ethiopia; 9 International Medical Corps, Country Office, Khartoum, Sudan

**Keywords:** Undernutrition, Good governance, Public health expenditures, Urbanization

## Abstract

**Background:**

Child undernutrition remains the major public health problem in low and middle-income countries including Ethiopia. The effects of good governance, urbanization and public health expenditure on childhood undernutrition are not well studied in developing countries. The objective of the study is to examine the relationship between quality of governance, public health expenditures, urbanization and child undernutrition in Ethiopia.

**Methods:**

This is pooled data analysis with ecological design. We obtained data on childhood undernutrition from the Ethiopian Demographic and Health Surveys (EDHS) that were conducted in 2000, 2005, 2011 and 2016. Additionally, data on quality of governance for Ethiopia were extracted from the World Governance Indicators (WGI) and public health spending and urbanization were obtained from the World Development Indicators and United Nations’ World Population Prospects (WPP) respectively. Univariate and multivariate analysis were done to assess the relationship between governance, public health expenditure and urbanization with childhood undernutrition.

**Result:**

Government effectiveness (adjusted odd ratio (AOR) = 20.7; *p* = 0.046), regulatory quality (AOR = 0.0077; *p* = 0.026) and control of corruption (AOR = 0.0019; *p* = 0.000) were associated with stunting. Similarly, government effectiveness (AOR = 72.2; *p* = 0.007), regulatory quality (AOR = 0.0015; *p* = 0.004) and control of corruption (AOR = 0.0005; *p* = 0.000) were associated with underweight. None of the governance indicators were associated with wasting. On the other hand, there is no statistically significant association observed between public health spending and urbanization with childhood undernutrition. However, other socio-demographic variables play a significant effect on reducing of child undernutrition.

**Conclusion:**

This study indicates that good governance in the country plays a significant role for reducing childhood undernutrition along with other socio-demographic factors. Concerned bodies should focus on improving governance and producing a quality policy and at the same time monitor its implementation and adherence.

**Electronic supplementary material:**

The online version of this article (10.1186/s12913-018-3822-2) contains supplementary material, which is available to authorized users.

## Background

Child undernutrition remains one of the major challenges in low-income and middle-income countries [1]. UNICEF’s conceptual frame underpin the cause of undernutrition as basic (comprises of social, economic, and political context that leads to lack of capital: financial, human, physical, social, and natural) and underlying (income poverty: employment, self-employment, dwelling, assets, remittances, pensions, transfers etc.) wherein poverty plays the central divisive role [1]. The resultant outcome for undernutrition is shown in different spectrums in the course of childhood development. Although a lot of factors are intertwined to affect child undernutrition, quality of governance and government commitment and action could contribute as fundamental elements in this regard.

In a given country, development as a whole and economic development in particular determines public health spending, and that in turn can play a vital role for improvement in health [2]. However, there is little evidence to suggest that increased spending contributes to meaningful reductions in health disparities [3]. The effect of public health spending can further be enhanced by good governance, and efficient allocation of public health spending can reduce child mortality [2, 4].

The essentiality of improving quality of governance through reduced corruption and maladministration is likely to result in efficient use of resources in the health sector [5]. Studies showed that governance is central element in determining the efficacy of public spending. In particular, a study by Rajkumar and Swaroop (2008) found out that, a 1% increase in the share of public health spending in gross domestic product (GDP) reduces under-5 mortality rate by 0.32% in countries with good governance and 0. 20% in countries with average governance, and has no impact for countries with weak governance [2]. This study pointed malpractice in governance as the cause of inefficiency in health service delivery system. In those countries with weak governance, public resources are washed out and are not converted into public investment. As a result, such poor quality of governance in countries limit the efficiency of conversion of public investment in to improved education and health services. In this regard, some literatures revealed that there is a link between good governance and lower mortality, and longer life expectancy [2, 6–8].

In addition to the role of improved health as an important source of productivity growth and economic development, it is the right of the citizen. Furthermore, adequate access to health services is a developmental milestone in order to attain the universal health coverage goals. Hence, the amount of resources in the health sector, as measured by public spending on health (PSH), is a potentially important determinant along with quality of governance [7].

Understanding how governance affects child health in the context of urbanization and economic development, is essential [9]. The same translation is expected in the country where there is economic growth with rapid expansion in urbanization. So far, there are limited studies carried out in developing countries regarding good governance and public health spending on child nutritional status. Therefore, this study examined the effect of governance quality, public health spending and urbanization on child undernutrition in Ethiopia given the partial effect of demographic and other economic covariates.

## Methods

We obtained the data from the Ethiopia Demographic and Health Surveys (EDHS) conducted by Central Statistical Agency (CSA) and ICF International provided technical assistance as well as funding to the project through the MEASURE DHS project. The DHS provide publicly available datasets designed to monitor health status in over 70 countries with large sample sizes (usually between 5000 and 30,000 households) and typically are conducted about every 5 years, to allow comparisons over time. The DHS program was established by the United States Agency for International Development (USAID) in 1984 [10]. The study used the publicly available DHS database and follows an in-depth secondary data analysis, form the period of 2000 to 2016 that offered available data for analyses. The nature of the survey was a national population based cross sectional study design that was conducted in a representative sample of women with reproductive age and their under five children in eleven regional states of Ethiopia [11].

The measure used to indicate child health outcome was child undernutrition expressed as stunting, wasting and underweight. The definition used were as follows: stunted (height-for age) - children whose height-for-age Z-score is below minus two standard deviations (− 2 SD) from the median of the reference population, underweight (weight-for age)-children under age five whose weight-for-age is below minus-two standard deviations from the median of the reference population and wasted (weight-for height) -children under age five whose weight-for-height is below minus-two standard deviations from the median of the reference population.

This study used World Governance Indicators (WGIs)^1^ [12] extracted from World Bank database. The indicators consist of six dimensions of good governance: voice and accountability, political stability and absence of violence, government effectiveness, regulatory quality, rule of law, and control of corruption. To measure the quality of governance in this study, we include all the items recommended by World Bank (WB).

The measurement of WGIs is scaled as a score (in standard deviations) from − 2.5 to 2.5 z-scores or zero and 100 and higher score suggests better outcomes for that country. We also employed public health spending (measured as a share or percentage of GDP contributed to the health sector) extracted from the World Development Indicators for the country at each particular year of survey [12] and urbanization (was measured as the proportion of a country’s population living in areas classified as urban, according to the criteria stated by a given country) extracted from the interpolated United Nations’ World Population Prospects (WPP) [13][Additional file 1].

The quantitative data of the three data sets were checked for completeness and consistency. Data analysis was carried out using STATA 14 (Stata Corporation, College Station, TX). The analysis included descriptive analysis about characteristics of the study population, and several of the variables used in the study.

This paper analyzes the effects of governance indicators, public health expenditure and urbanization on child undernutrition. We use univariate and multivariate analysis to assess the relationship. In order to control heteroskedasticity, we use multivariate regression model with robust estimation of standard errors. This multivariate regression model was fitted to determine the associations between the categorized variables of childhood undernutrition after controlling for several potentially confounding factors. At the final model, those variables that retained a *p* < 0.05 was considered to be statistical significant and used to interpret the finding of the study. Ethical approval for this study was not sought due to the secondary analysis of publicly available aggregate data. However, the DHS follows a standard protocol given and approved by the Ethiopia Health and Nutrition Research Institute (EHNRI) Review Board, the National Research Ethics Review Committee (NRERC) at the Ministry of Science and Technology, the Institutional Review Board of ICF International, and the CDC.

## Results

From our datasets, sampled 43,029 observations over four years (2000, 2005, 2011 and 2016) (see Table 1) were used. On average, the finding demonstrate that the overall mean governance scores were voice and accountability (− 1.18), political stability and absence of violence (− 1.08), government effectiveness (− 0.71), regulatory quality (− 1.07), rule of law (− 0.73), control of corruption (− 0.67), urbanization (16.2) and public health expenditures (12.3). Table 2 shows the overall correlation among the variables of interest. All these predictors are significantly correlated, with *p*-values of < 0.001. High correlations are observed between the governance indicators (Pearson’s correlation coefficient, *r* = 0.49–0.99 with *p* < 0.001). In the same way, there is a high correlation between urbanization and public health expenditures (*r* = 0.98, *p* < 0.001).

**Table 1:** Summary statistics of good governance, urbanization and public health expenditures

Variables	n	Mean	SD	Minimum	Median	Maximum
Good governance						
Voice and accountability	43,029	−1.18	0.09	−1.28	−1.24	− 1,03
Political stability and absence of violence	43,029	−1.08	0.11	−1.23	−1.06	−0.92
Government effectiveness	43,029	−0.71	0 .24	−1.04	−0.51	− 0.46
Regulatory quality	43,029	−1.07	0.12	−1.23	−1.05	−0.90
Rule of law	43,029	−0.73	0.12	−0.85	−0.68	− 0.56
Control of corruption	43,029	−0.67	0.09	−0.78	−0.67	− 0.52
Urbanization	43,029	16.2	1.68	14.3	16.5	18.8
Public Health expenditures	43,029	12.3	3.72	7.54	13.9	17.3

**Table 2:** Pearson correlation between good governance, urbanization and public health expenditures

Variables*	1	2	3	4	5	6	7	8
(1) Voice and accountability	1.00							
(2) Political stability and absence of violence	−0.81	1.00						
(3) Government effectiveness	−0.94	0.92	1.00					
(4) Regulatory quality	−0.81	0.99	0.93	1.00				
(5) Rule of law	−0.86	0.63	0.87	0.64	1.00			
(6) Control of corruption	−0.87	0.49	0.78	0.50	0.95	1.00		
(7) Urbanization	−0.90	0.56	0.83	0.57	0.97	0.99	1.00	
(8) Public health expenditures	−0.94	0.70	0.92	0.70	0.98	0.95	0.98	1.00

* *P*-values for all correlations < 0.001

### Factors associated with stunting, wasting and underweight

Table 3 shows that there was continuous reduction in the prevalence probability to child undernutrition problem in Ethiopia in the last two decades. The overall rate of change reveals that the rate of change for underweight problem was highest compared stunting and wasting in the previous four surveys. Meanwhile, our analysis confirms that all the dimensions included in measuring good governance are significantly associated with child undernutrition. Government effectiveness^2^ (AOR = 20.7; *p* = 0.046), regulatory quality (AOR = 0.0077; *p* = 0.026) and control of corruption (AOR = 0.0019; *p* = 0.000) were associated with stunting. This indicate that improvement in the government effectiveness, regulatory quality and control of corruption would significantly reduce the prevalence to child stunting in Ethiopia. Similarly, government effectiveness (AOR = 72.2; *p* = 0.007), regulatory quality (AOR = 0.0015; *p* = 0.004) and control of corruption (AOR = 0.0005; *p* = 0.000) had significant association with children underweight. None of the governance indicators had significant association with wasting. Our finding also showed that there is no statistically significant association between public health spending and urbanization with childhood undernutrition in Ethiopia. However, other socio-demographic factors play a significant role on child undernutrition in the country (Tables 4, 5 & 6). Sex of the child, current age of child, presence of more than 2 under five children at home, partner’s education level, wealth index, improved/modern sanitation type of toilet, improved source of drinking water, mothers’ education level, parity, and age of the mother, region had significant interaction with child stunting (Table 4). In the same line, sex of the child, partner’s education level, current age of child, wealth index, mothers’ educational level, parity more than 3, improved/modern sanitation and region had significant association with wasting (Table 5). Moreover, explanatory variables such as sex of the child, partner’s education level, current age of child, wealth index, mothers’ education, age of the mother, parity more than 3, improved/modern sanitation, presence of more than 2 under five children at home, residence, and region were significantly associated with underweight (Table 6).

**Table 3:** Prevalence probability to child undernutrition in Ethiopia

Undernutrition	Survey year				Rate of change (from 2000 to 2016)
	2000(*n* = 10,873)	2005(*n* = 9861)	2011(*n* = 11,654)	2016(n = 10,641)	
Stunting	53.30	46.95	42.11	34.20	35.84
Underweight	39.65	33.12	30.41	23.70	40.23
Wasting	13.88	13.07	12.14	12.15	12.46

Note: values in the table are in percentage

**Table 4:** Multivariate Regression Models of good governance and other independent variables on stunting among children age 6–59 months in Ethiopia

Variables		Model_1			Model_2		
		Odds ratio	R. Sta. err	*p*-value	Odds ratio	R. Sta. err	*p*-value
Government effectiveness		3.90	5.75	0.354	20.7	31.46	0.046
Regulatory quality		0.09	0.19	0.252	0.0077	0.0168	0.026
Control corruption		0.02	0.02	0.009	0.0019	0.0032	0.000
Region (Tigray region is ref.)	Affar				0.95	0.076	0.542
	Amhara				1.14	0.08	0.054
	Oromia				0.79	0.05	0.000
	Somali				0.592	0.048	0.000
	Beni-Gumuz				0.915	0.069	0.244
	SNNP				0.968	0.062	0.618
	Gambela				0.519	0.048	0.000
	Harari				0.601	0.054	0.000
	Addis Ababa				0.6710	0.076	0.000
	Dire Dawa				0.624	0.061	0.000
Sex (Male is ref.)	Female				0.876	0.022	0.000
Partner’s education level (Ref. No education)	Primary				0.924	0.031	0.020
	Secondary				0.711	0.045	0.000
	Higher				0.622	0.567	0.000
Current age of child (0 years is ref.)	1 years				4.44	0.192	0.000
	2 years				6.39	0.279	0.000
	3 years				5.851	0.264	0.000
	4 years				4.706	0.219	0.000
Wealth index Quintile (Poorest is ref.)	Poorer				0.952	0.042	0.267
	Middle				0.904	0.040	0.023
	Richer				0.842	0.037	0.000
	Richest				0.746	0.041	0.000
Toilet type (Unimproved is ref.)	Improved/modern sanitation				0.825	0.041	0.000
Drinking source (Unimproved is ref.)	Improved source of drinking water				0.905	0.042	0.031
Residence (Urban is ref.)	Rural				1.117	0.077	0.109
Highest educational level of the respondents (illiterate is ref.)	Primary				0.918	0.035	0.027
	Secondary				0.595	0.050	0.000
	Higher				0.447	0.077	0.000
Number of children under five (≤ 2 is ref.)	> 2				0.879	0.036	0.002
Sex of household head (Male is ref.)	Female				1.065	0.042	0.108
Respondent’s occupation (Not working is ref.)	Paid working				1.055	0.042	0.175
	Agricultural				1.043	0.041	0.279
Partner’soccupation (Not working is ref.)	Paid working				1.102	0.084	0.201
	Agricultural				1.131	0.080	0.082
Parity (parity≤3 is ref.**)**	Parity more than 3				1.133	0.046	0.002
Age of women (15–19 is the ref.)	20–24				0.980	0.076	0.797
	25–29				0.926	0.076	0.347
	30–34				0.890	0.075	0.167
	35–39				0.863	0.075	0.091
	40–44				0.768	0.075	0.007
	45–49				0.680	0.078	0.001
Number of householdmembers (1–3 members is ref.)	4–6 members				0.960	0.047	0.405
	> 7 members				0.977	0.055	0.681
R^2^		0.0160			0.1128

**Table 5:** Multivariate Regression Models of good governance and other independent variables on wasting among children age 6–59 months in Ethiopia

Variables		Model_1			Model_2		
		Odds ratio	R. Sta. err	*P*-value	Odds ratio	R. Sta. err	*P*-value
Government effectiveness		1.364	2.774	0.879	0.768	1.660	0.903
Regulatory quality		0.449	1.313	0.784	0.967	3.007	0.991
Control corruption		0.514	1.126	0.761	0.710	1.663	0.884
Region (Tigray region is ref.)	Affar				1.702	0.164	0.000
	Amhara				1.000	0.083	0.998
	Oromiya				0.892	0.074	0.166
	Somali				1.94	0.184	0.000
	Beni-Gumuz				1.186	0.114	0.077
	SNNP				0.983	0.083	0.842
	Gambela				1.504	0.170	0.000
	Harari				0.790	0.119	0.117
	Addis Ababa				0.572	0.112	0.004
	Dire Dawa				1.333	0.167	0.022
Sex (Male is ref.)	Female				0.803	0.028	0.000
Partner’s education level (Ref. No education)	Primary				0.875	0.030	0.000
	Secondary				0.783	0.050	0.000
	Higher				0.645	0.073	0.000
Current age of child (0 years is ref.)	1 years				0.872	0.043	0.006
	2 years				0.529	0.029	0.000
	3 years				0.370	0.022	0.000
	4 years				0.447	0.026	0.000
Wealth index Quintile (Poorest is ref.)	Poorer				0.908	0.052	0.089
	Middle				0.895	0.052	0.058
	Richer				0.806	0.048	0.000
	Richest				0.779	0.054	0.000
Toilet type (Unimproved is ref.)	Improved/modern sanitation				0.827	0.059	0.008
Drinking source (Unimproved is ref.)	Improved source of drinking water				0.975	0.061	0.678
Residence (Urban is ref.)	Rural				0.908	0.803	0.292
Highest educational level of the respondents (illiterate is ref.)	Primary				0.885	0.052	0.036
	Secondary				0.668	0.080	0.001
	Higher				0.604	0.135	0.024
No of children under five (≤ 2 is ref.)	> 2				0.993	0.049	0.892
Sex of household head (Male is ref.)	Female				1.084	0.057	0.123
Respondent’s occupation (Not working is ref.)	Paid working				0.953	0.052	0.497
	Agricultural				1.069	0.055	0.378
Partner’soccupation (Not working is ref.)	Paid working				0.859	0.083	0.196
	Agricultural				0.994	0.089	0.945
Parity (parity≤3 is ref.)	Parity more than 3				1.141	0.067	0.024
Age of women (15–19 is the ref.)	20–24				1.008	0.092	0.930
	25–29				0.908	0.086	0.311
	30–34				0.989	0.099	0.919
	35–39				0.952	0.100	0.639
	40–44				1.083	0.122	0.481
	45–49				0.919	0.139	0.577
Number of householdmembers (1–3 members is ref.)	4–6 members				0.986	0.073	0.847
	> 7 members				0.948	0.079	0.521
R^2^		0.0007			0.0462

**Table 6:** Multivariate Regression Models of good governance and other independent variables on underweight among children age 6–59 months in Ethiopia

Variables		Model_1			Model_2		
		Odds ratio	R. Sta. err	*P*-value	Odds ratio	R. Sta. err	*P*-value
Government effectiveness		17.31	28.65	0.085	72.2	114.9	0.007
Regulatory quality		0.012	0.028	0.062	0.0015	0.003	0.004
Control corruption		0.004	0.006	0.002	0.0005	0.0009	0.000
Region (Tigray region is ref.)	Affar				1.135	0.117	0.001
	Amhara				1.07	0.076	0.311
	Oromiya				0.810	0.056	0.002
	Somali				1.044	0.090	0.619
	Beni-Gumuz				1.145	0.094	0.100
	SNNP				1.002	0.699	0.972
	Gambela				0.730	0.067	0.001
	Harari				0.574	0.066	0.000
	Addis Ababa				0.475	0.061	0.000
	Dire Dawa				0.894	0.092	0.275
Sex (Male is ref.)	Female				0.867	0.023	0.000
Partner’s education level (Ref. No education)	Primary				0.875	0.030	0.000
	Secondary				0.783	0.050	0.000
	Higher				0.645	0.073	0.000
Current age of child (0 years is ref.)	1 years				2.389	0.106	0.000
	2 years				2.704	0.116	0.000
	3 years				2.408	0.109	0.000
	4 years				2.471	0.113	0.000
Wealth index Quintile (Poorest is ref.)	Poorer				0.888	0.038	0.006
	Middle				0.857	0.037	0.000
	Richer				0.754	0.036	0.000
	Richest				0.716	0.043	0.000
Toilet type (Unimproved is ref.)	Improved/modern sanitation				0.791	0.042	0.000
Drinking source (Unimproved is ref.)	Improved source of drinking water				0.932	0.050	0.196
Residence (Urban is ref.)	Rural				1.25	0.102	0.007
Highest educational level of the respondents (illiterate is ref.)	Primary				0.853	0.037	0.000
	Secondary				0.572	0.056	0.000
	Higher				0.366	0.084	0.000
No of children under five (≤ 2 is ref.)	> 2				0.844	0.034	0.000
Parity (parity≤3 is ref.**)**	Parity more than 3				1.24	0.049	0.000
Sex of household head (Male is ref.)	Female				1.056	0.043	0.178
Respondent’s occupation (Not working is ref.)	Paid working				0.990	0.044	0.380
	Agricultural				1.012	0.040	0.770
Partner’s occupation (Not working is ref.)	Paid working				1.022	0.082	0.782
	Agricultural				1.088	0.085	0.280
Age of women (15–19 is the ref.)	20–24				0.976	0.082	0.773
	25–29				0.973	0.084	0.750
	30–34				1.00	0.089	0.999
	35–39				0.914	0.086	0.336
	40–44				0.900	0.091	0.300
	45–49				0.769	0.095	0.033
No of household members (ref. is 1–3 members)	4–6 members				0.964	0.051	0.495
	> 7 members				0.934	0.056	0.254
R^2^		0.0137			0.0744		

## Discussion

There is abundant literature that examines the household related factors that are associated with child undernutrition. The literature becomes scarce when it comes to the relationship between societal (or state) related factors and child undernutrition. This paper focuses on these state and macro-economic factors by giving emphasis on quality of governance, public health expenditure and urbanization, and their implication on child undernutrition.

In this study, governance (government effectiveness, control of corruption and regulator quality) is one of the major factors associated with child undernutrition in Ethiopia. The role of good governance to reduce child undernutrition appears substantial in this study, and we confirm that it has significant contribution to reduce child stunting and underweight in Ethiopia. Similar to our study, good governance significantly reduce the under-five mortality rates (U5MR) in the worst five countries with regards to U5MR [8].

Such positive contribution of good governance to reduce child undernutrition brought the agenda at the top frontline. There is an increased recognition that the ability of governments to be responsive and responsible is crucial to bring a profound influence on a number of factors that determine nutrition status [14]. Similarly, Smith and Haddad (15) suggested increased investment to improve governance, in addition to key infrastructure development investments, in order to accelerate reductions in undernutrition. The finding from a global project of dataset of 96 countries, which comprises 91% of the global population, shows that the higher the country’s level of adherence to the rule of law, the better the population health would be. The conclusion is that poor progress in rule of law may constitute a structural barrier to health improvement in the country [15].

The overall aspect of having good governance is to bring an effective strategy that enables the society to exercise its right and adhere with its responsibility and participation in the country utmost effort to realize the country ownership and development endeavor. In a new study revealed out from 139 low and middle-income countries (LMICs), only 39.6% of them had policies to address both undernutrition and overweight/obesity. Strong nutrition governance in LMICs was associated with low magnitudes of stunting, wasting and underweight [16].

Healthy and a well-developed population are more energetic and productive to the nation. Good governance in the country in general and in the health sector in particular can contribute towards this objective. Having a good policy alone, unless supported with a well-functioning governance structure to implement the policy, cannot guarantee for maintaining health and nutrition of the population. In most of the developing countries, lack of clear nutrition policies together with poor governance hinder attempts to address the dual burdens of malnutrition. These also explain some of the slow progress made so far in these countries in addressing childhood undernutrition [17].

Moreover, according to Eran Bendavid (2014), in a study encompassing 54 countries between 1995 and 2012 found a relationship between under-5 mortality rate reduction and slow progress in good governance in LMICs [18]. Likewise, good governance can be important for improving equity in child mortality, at least in LMICs [19]. Existing studies argue that governance matters for child survival through the channel of providing public goods, including public health interventions [20, 21]. In this regard, a study carried out in 149 countries between 1996 and 2010 showed that countries with better governance are likely to have health policies that benefit the social determinants of health [22]. By combining governance and public health spending, Rajkumar and Swaroop (2008) report the variation on the effect of PSH across different governance qualities. They indicated that 1% increment in the share of PSH from the GDP lowers under-five mortality rates (U5MR) rate by 0.32% in countries with good governance, 0.20% in countries with average governance, and has no impact in countries with weak governance [2]. Such a variation on the effect of PSH across governance qualities is potentially associated with the inefficiency of governments to convert public health spending into key health and nutrition outcomes. Similarly, a study using data from 110 countries for the years between 1996 and 2007 indicated that corruption causes a decrease in genuine wealth per person, and this implies that corruption affects sustainable development negatively [23].

Urbanization is another factor that is associated with child health and nutrition outcome. Our model result did not demonstrate a significant association between urbanization and child undernutrition. According to Tord and Susan (2008), better housing and living conditions, access to safe water and good sanitation, efficient waste management systems, safer working environments and neighborhoods, food security and access to services such as education, health, welfare, public transportation and child care are social determinants of health that can be addressed through good urban governance. In this way, a failure of governance in the cities has resulted in the growth of informal settlements and slums that constitute unhealthy living and working environments for one billion people [24]. And this has a negative implication on health and nutrition in the developing world.

A study done in Nigeria showed that urbanization has significantly negative relationship with health sector; however, the lagged value of the population living in the urban areas according to Urban Populations Outreach Program (UPOP) is positively related to infant mortality rate (IMR) and U5MR. This is not surprising in a country with poor institutional facilities. Such a system can encourage rent seeking behavior and poor enforcement of rule of law, and may discourage long term planning with respect to urban planning and provision of social infrastructures to match population growth rate and government efficiency [25]. The result of government spending and increasing urbanization accompanied with proper urban planning and rule of law through quality institutions can have positive health and human capacity development outcomes. Conversely, the absence of such an integrated system will result in deteriorating health status of the citizens and poor human capital development [25]. Evidence documented that there are interplays on the factors that affect a given health outcomes. In a certain circumstance, income influences health through improved nutrition, housing, and access to water and sanitation and literacy as a few exemplified points for our argument [4]. The study has some strengths and limitations of this study. Several factors where elucidated such as good governance, urbanization, public health spending and other socio-economic variables that explain under nutrition in the country and the research was based on highly representative pooled data. However, this study has a limitation of a cross-sectional design that should be interpreted cautiously due to difficulty to distinguish cause-and-effect relationships and reporting and recall bias should be considered as some of the events asked retrospectively.

In conclusion, this study indicates that good governance in the country can play a significant role for childhood undernutrition along with other socio-demographic parameters. It is vital to stress the fact that countries require integrated nutrition and health policies and good governance to effectively address all the dimensions of child undernutrition. Concerned bodies should focus on producing a quality policy and monitoring its proper implementation adherence for its policies and strategies.This paper also highlighted the important role of the private sector in the reduction of child undernutrition.

## Additional file

Additional file 1: Appendix 1: Dimensions of parameters. (DOCX 14 kb)

## Abbreviations

AOR: Adjusted odd ratio
CSA: Central Statistical Agency
EDHS: Ethiopia demographic and health surveys
GDP: Gross domestic product
LMICs: Low and middle-income countries
PSH: Public spending on health
U5MR: Under-five mortality rates
UNICEF’s: The United Nations International Children’s Emergency Fund
UNWPP: United nations’ world population prospects
USAID: United States Agency for International Development (USAID)
WGIs: World Governance Indicators

## Footnotes

1 Definitions with elaborations are given in the annex

2 Since the values of good governance (government effectiveness, regulatory quality and control of corruption) for Ethiopia are negative, then interpretation of the coefficients should be in the opposite direction.
