# Supplementary material for: Good governance, public health expenditures, urbanization and child undernutrition Nexus in Ethiopia: an ecological analysis
Source: BMC Health Serv Res. 2019 Jan 15;19:40. doi: 10.1186/s12913-018-3822-2 (PMC6334413; doi:10.1186/s12913-018-3822-2)
Supplement: Supplementary file 1 — Appendix 1: Dimensions of parameters. (DOCX 14 kb) [file 12913_2018_3822_MOESM1_ESM.docx]

**Appendix 1: Dimensions of parameters**

| **Parameters** | **Description** |
| --- | --- |
| Voice and accountability | Perceptions of the extent to which a country’s citizens are able to participate in selecting their government, as well as freedom of expression, freedom of association, and a free media. It ranges from approximately -2.5 (weak) to 2.5 (strong) (World Bank’s World Governance Indicators [WGI] |
| Political stability and absence of violence | Perceptions of the likelihood that the government will be destabilized or overthrown by unconstitutional or violent means, including politically motivated violence and terrorism. It ranges from approximately -2.5 (weak) to 2.5 (strong) (World Bank’s WGI). |
| Government effectiveness | People perception about the quality of public services, civil service and policy formulation and implementation. It also includes the degree of its independence from political pressures, and credibility of the government's commitment to such policies. It ranges from approximately -2.5 (weak) to 2.5 (strong) (World Bank’s WGI). |
| Regulatory quality | People perceptions about the ability of the government to formulate and implement sound policies and regulations that permit and promote private sector development. It ranges from approximately -2.5 (weak) to 2.5 (strong). It ranges from approximately -2.5 (weak) to 2.5 (strong) (World Bank’s WGI). |
| Control of corruption | People perceptions on the extent to which public power is exercised for private gain, including both petty and grand forms of corruption, as well as "capture" of the state by elites and private interests. It ranges from approximately -2.5 (weak) to 2.5 (strong) (World Bank’s WGI). |
| Rule of law | Perceptions of the extent to which agents have confidence in and abide by the rules of society, and in particular the quality of contract enforcement, property rights, the police, and the courts, as well as the likelihood of crime and violence. It ranges from approximately -2.5 (weak) to 2.5 (strong) (World Bank’s WGI). |
| Urbanization | It was measured as the proportion of the population living in urban areas. Collected from United Nations’ World Population Prospects (United Nations’ World Population Prospects25, United Nations. World Population Prospects: The 2010 Revision. http://esa.un.org/wpp/ |
| Public health expenditures | It is the public expenditure (the local government) for health. Sum of public and private health expenditures as a percentage of total GDP, which covers the provision of health services (preventive and curative), family planning activities, nutrition activities, and emergency aid designated for health but does not include provision of water and sanitation. Measured as a share or percentage of GDP contributed to the health sector. It was just plain overall spending, but not per capita/child public health spending (World Bank’s WDI The World Bank. World Development Indicators (WDI). |

Note: Values of all the parameters were average for five years preceding each survey

Since the dependent variables (stunting, underweight and wasting) are for children under five, the parameters to measure good governance and development should be parallel in year, and ought to be computed average for previous five years, proceeding each survey year. Yearly values of good governance and development indicators could not measure the five-year problems (stunting, underweight and wasting) of children under investigation. This was the logic behind computing five-year average values for good governance and development parameters.
